# Physical activity and sedentary behaviour in relation to cardiometabolic risk in children: cross-sectional findings from the Physical Activity and Nutrition in Children (PANIC) Study

**DOI:** 10.1186/1479-5868-11-55

**Published:** 2014-04-26

**Authors:** Juuso Väistö, Aino-Maija Eloranta, Anna Viitasalo, Tuomo Tompuri, Niina Lintu, Panu Karjalainen, Eeva-Kaarina Lampinen, Jyrki Ågren, David E Laaksonen, Hanna-Maaria Lakka, Virpi Lindi, Timo A Lakka

**Affiliations:** 1 Institute of Biomedicine, Physiology, University of Eastern Finland, Kuopio, Finland; 2 Institute of Dentistry, School of Medicine, University of Eastern Finland, Kuopio, Finland; 3 Department of Clinical Physiology and Nuclear Medicine, Kuopio University Hospital, Kuopio, Finland; 4 Institute of Clinical Medicine, Internal Medicine, Kuopio University Hospital, Kuopio, Finland; 5 Kuopio Research Institute of Exercise Medicine, Kuopio, Finland

**Keywords:** Child, Metabolic diseases/metabolism, Motor activity/physiology, Risk factors, Sedentary lifestyle

## Abstract

**Background:**

Lower levels of physical activity (PA) and sedentary behaviour (SB) have been associated with increased cardiometabolic risk among children. However, little is known about the independent and combined associations of PA and SB as well as different types of these behaviours with cardiometabolic risk in children. We therefore investigated these relationships among children.

**Methods:**

The subjects were a population sample of 468 children 6–8 years of age. PA and SB were assessed by a questionnaire administered by parents and validated by a monitor combining heart rate and accelerometry measurements. We assessed body fat percentage, waist circumference, blood glucose, serum insulin, plasma lipids and lipoproteins and blood pressure and calculated a cardiometabolic risk score using population-specific Z-scores and a formula waist circumference + insulin + glucose + triglycerides - HDL cholesterol + mean of systolic and diastolic blood pressure. We analysed data using multivariate linear regression models.

**Results:**

Total PA was inversely associated with the cardiometabolic risk score (β = -0.135, p = 0.004), body fat percentage (β = -0.155, p < 0.001), insulin (β = -0.099, p = 0.034), triglycerides (β = -0.166, p < 0.001), VLDL triglycerides (β = -0.230, p < 0.001), VLDL cholesterol (β = -0.168, p = 0.001), LDL cholesterol (β = -0.094, p = 0.046) and HDL triglycerides (β = -0.149, p = 0.004) and directly related to HDL cholesterol (β = 0.144, p = 0.002) adjusted for age and gender. Unstructured PA was inversely associated with the cardiometabolic risk score (β = -0.123, p = 0.010), body fat percentage (β = -0.099, p = 0.027), insulin (β = -0.108, p = 0.021), triglycerides (β = -0.144, p = 0.002), VLDL triglycerides (β = -0.233, p < 0.001) and VLDL cholesterol (β = -0.199, p < 0.001) and directly related to HDL cholesterol (β = 0.126, p = 0.008). Watching TV and videos was directly related to the cardiometabolic risk score (β = 0.135, p = 0.003), body fat percentage (β = 0.090, p = 0.039), waist circumference (β = 0.097, p = 0.033) and systolic blood pressure (β = 0.096, p = 0.039). Resting was directly associated with the cardiometabolic risk score (β = 0.092, p = 0.049), triglycerides (β = 0.131, p = 0.005), VLDL triglycerides (β = 0.134, p = 0.009), VLDL cholesterol (β = 0.147, p = 0.004) and LDL cholesterol (β = 0.105, p = 0.023). Other types of PA and SB had less consistent associations with cardiometabolic risk factors.

**Conclusions:**

The results of our study emphasise increasing total and unstructured PA and decreasing watching TV and videos and other sedentary behaviours to reduce cardiometabolic risk among children.

**Trial registration:**

ClinicalTrials.gov Identifier: NCT01803776.

## Background

Overweight and obesity among children are a major public health problem in developed countries including Finland [1-3]. The epidemic of childhood obesity has also increased the prevalence of paediatric metabolic syndrome [4-6]. Moreover, overweight and obesity track from childhood into adulthood and are associated with an increased risk of metabolic syndrome, type 2 diabetes and cardiovascular disease in adulthood [7,8]. Therefore, the prevention of clustering of metabolic risk factors should commence in early childhood.

The metabolic syndrome represents a cluster of cardiometabolic abnormalities, including abdominal obesity, insulin resistance, glucose intolerance, dyslipidaemia and elevated blood pressure [4,9]. The definitions of paediatric metabolic syndrome have been criticised, because they are based on artificial cut-offs for the individual features of metabolic syndrome used in adults [10,11]. Therefore, studies among children or adolescents have often employed composite cardiometabolic risk scores that have been calculated using the features of metabolic syndrome as continuous variables [12-15].

Lower levels of physical activity (PA) and higher levels of sedentary behaviour (SB) have been associated with an increased overall cardiometabolic risk [12,13,16,17] and overweight [18-20] among children. However, there are few studies on the independent associations of PA and SB with cardiometabolic risk factors other than overweight in children [19,21]. A recent study showed that lower levels of PA were associated with many cardiometabolic risk factors independent of SB and that watching TV, playing video games and using computer were related to some cardiometabolic risk factors independent of PA among children [21]. Moreover, little is known about the combined associations of PA and SB with cardiometabolic risk factors [21]. Whereas watching TV, playing video games and using computer have been associated with overweight among children, there are no studies on the independent relationships of other types of PA or SB with clustered cardiometabolic risk or individual cardiometabolic risk factors among children.

We investigated the independent and combined associations of PA and SB as well as different types of these behaviours with overall cardiometabolic risk using cardiometabolic risk score and with individual cardiometabolic risk factors.

## Methods

### Study design and study population

The present study is based on the baseline data from the Physical Activity and Nutrition in Children (PANIC) Study, which is an ongoing exercise and diet intervention study in a population sample of primary school children from the city of Kuopio, Finland. Altogether 736 children aged 6–8 years who started first grade in primary schools in 2007–2009 were invited to participate in the baseline examinations between October 2007 and November 2009. Of the invited children, 512 (70%) participated. Complete data on variables used in the analyses were available for 468. The study protocol was approved by the Research Ethics Committee of the Hospital District of Northern Savo. All participating children and their parents gave informed written consent.

### Assessment of physical activity and sedentary behaviour

Habitual PA during a usual week was assessed by the PANIC Physical Activity Questionnaire administered by the parents at home. The types of PA included organised sports, structured exercise, unstructured PA, commuting to and from school and PA during recess. The frequency of each type of PA, expressed in sessions per week, and the duration of a single session of each type of PA, expressed in hours per session to accuracy of half an hour, were asked. The amount of each PA type was calculated by multiplying frequency with duration and was expressed in minutes per day. The amount of total PA was calculated by summing the amounts of each PA type and was expressed in minutes per day. All children in the first grade had 90 minutes of physical education per week that was included in total PA.

Habitual SB during usual five weekdays and two weekend days was also assessed by the PANIC Physical Activity Questionnaire administered by the parents at home. The types of SB included watching TV and videos, using a computer and playing video games, using a mobile phone and playing mobile games, listening to music, playing a musical instrument, reading, writing, drawing, doing arts and crafts, playing board games and resting. Electronic media time (EMT) was calculated by summing watching TV and videos, using a computer and playing video games and using a mobile phone and playing mobile games. The amount of total SB was calculated by summing the times spent in each SB and was expressed in minutes per day weighted by the number of weekdays and weekend days.

We validated the PANIC Physical Activity Questionnaire using the Actiheart monitor combining heart rate and accelerometry measurements (Actiheart, CamNtech, Cambridge, UK) in a subsample of 38 children examined at baseline of the PANIC Study. Total PA measured by the questionnaire correlated positively with total PA measured by the Actiheart monitor (r = 0.37, p = 0.033).

### Assessment of dietary factors

The number of meals and snacks per day, food consumption and nutrient intake were assessed by food records on four consecutive days [22].

### Assessment of cardiometabolic risk factors

Body composition and blood pressure were assessed by a research nurse at Institute of Biomedicine, University of Eastern Finland. Body height was measured by a wall-mounted stadiometer and body weight by the Inbody 720® bioimpedance device [23]. Body mass index (BMI) was calculated as body weight divided by body height squared. BMI-standard deviation score (BMI-SDS) was assessed by national references [24]. Body fat percentage and lean mass were measured by the Lunar® dual energy x-ray absorptiometry (DXA) device [23]. Waist circumference was measured after expiration at mid-distance between the bottom of the rib cage and the top of the iliac crest [23]. Blood pressure was measured manually on the right arm by a calibrated aneroid sphygmomanometer (Heine 130 Gamma G7, Munich, Germany). The measurement protocol included, after a rest of five minutes, three measurements in the sitting position at 2-minute intervals. The mean of all three values were used as the systolic and diastolic blood pressure.

Venous blood samples were taken after a 12-hour overnight fast by a laboratory nurse at Institute of Biomedicine, University of Eastern Finland. The blood samples were analysed at Laboratory of Clinical Chemistry at University Hospital of Kuopio. The assessment of plasma glucose, total, high-density lipoprotein (HDL) and low-density lipoprotein (LDL) cholesterol and triglycerides as well as serum insulin from 12-hour fasting samples has been explained previously [25]. Very low-density lipoprotein (VLDL) was separated by ultracentrifugation and HDL by precipitation of LDL after removal of VLDL fraction [26].

A continuous cardiometabolic risk score variable was calculated as the sum of Z-scores of waist circumference, insulin, glucose, triglycerides, HDL cholesterol and the mean of systolic and diastolic blood pressure that are specific for the PANIC study population. The Z-score of HDL cholesterol was multiplied by -1, because HDL cholesterol is inversely associated with cardiometabolic risk. A higher cardiometabolic risk score indicates a less favourable cardiometabolic risk profile.

### Statistical analyses

Statistical analyses were performed with the SPSS software, Version 21.0 (Armonk, NY: IBM Corp.). To normalise skewed distributions, a natural logarithmic transformation was performed for waist circumference and triglycerides, VLDL triglycerides, VLDL cholesterol and HDL triglycerides and a square-root transformation was performed for insulin. Differences in basic characteristics betweengenders were tested with the *T*-test for independent samples. The associations of different types of PA and SB with cardiometabolic risk score and individual cardiometabolic risk factors were analysed using linear regression models and standardised regression coefficients (β) adjusted for age and gender. Total PA and EMT were divided into three groups of similar size at tertiles of the distribution of these variables (thirds). Cardiometabolic risk score and individual cardiometabolic risk factors were compared across the thirds of PA and EMT using general linear models (GLM) adjusted for age and gender to explore possible nonlinear associations. The combined associations of PA and EMT with cardiometabolic risk factors were analysed by dichotomising PA and EMT at the median and comparing cardiometabolic risk factors in the four groups using GLM adjusted for age and gender. Associations with a P-value <0.05 were considered statistically significant.

## Results

### Basic characteristics

The girls had lower levels of PA and EMT, higher levels of most other types of SB, a poorer cardiorespiratory fitness and less favourable levels of most cardiometabolic risk factors than the boys (Table 1).

**Table 1:** Basic characteristics

	**All (N = 468)**	**Girls (N = 225)**	**Boys (N = 243)**	P-value for difference*
Age (years)	7.6 ± 0.4	7.6 ± 0.4	7.7 ± 0.4	0.180
Height (cm)	128.7 ± 5.7	127.6 ± 5.6	129.7 ± 5.5	**<0.001**
Weight (kg)	26.8 ± 5.7	26.4 ± 5.0	27.1 ± 4.7	0.113
Body mass index	16.1 ± 2.1	16.1 ± 2.2	16.1 ± 2.0	0.767
Body mass index standard deviation score^a^	-0.20 ± 1.1	-0.18 ± 1.1	-0.22 ± 1.1	0.662
Waist circumference (cm)	56.6 ± 5.5	55.9 ± 5.8	57.2 ± 5.2	**0.008**
Body fat percentage (%)	19.6 ± 8.1	22.4 ± 7.6	17.0 ± 7.7	**<0.001**
Fasting serum insulin (mU/I)	4.5 ± 2.5	4.8 ± 2.3	4.3 ± 2.6	**0.003**
Fasting plasma glucose (mmol/l)	4.8 ± 0.4	4.8 ± 0.4	4.9 ± 0.4	**0.004**
Fasting plasma triglycerides (mmol/l)	0.60 ± 0.24	0.62 ± 0.25	0.58 ± 0.23	0.052
Fasting plasma HDL cholesterol (mmol/l)	1.60 ± 0.31	1.57 ± 0.31	1.64 ± 0.32	**0.011**
Fasting plasma LDL cholesterol (mmol/l)	2.36 ± 0.51	2.41 ± 0.53	2.31 ± 0.49	**0.022**
Fasting plasma VLDL triglycerides (mmol/l)^b^	0.28 ± 0.20	0.30 ± 0.21	0.27 ± 0.19	0.272
Fasting plasma VLDL cholesterol (mmol/l)^b^	0.12 ± 0.10	0.13 ± 0.11	0.12 ± 0.10	0.466
Fasting plasma HDL triglycerides (mmol/l)^b^	0.16 ± 0.04	0.16 ± 0.04	0.15 ± 0.03	0.389
Fasting plasma total cholesterol (mmol/l)	4.27 ± 0.62	4.31 ± 0.63	4.23 ± 0.61	0.149
Systolic blood pressure (mmHg)	100.1 ± 7.3	99.8 ± 7.6	100.4 ± 7.1	0.418
Diastolic blood pressure (mmHg)	61.7 ± 6.6	61.5 ± 6.8	61.8 ± 6.5	0.629
Cardiometabolic risk score	-0.03 ± 3.51	0.02 ± 3.42	-0.08 ± 3.60	0.740
Total physical activity (min/d)^c^	111.4 ± 42	104.3 ± 39.1	117.9 ± 43.7	**<0.001**
Unstructured physical activity (min/d)	49.7 ± 31.5	44.6 ± 30.8	54.4 ± 31.4	**0.001**
Structured exercise (min/d)^d^	6.4 ± 11.3	5.0 ± 7.3	7.7 ± 13.9	**0.009**
Organised sports (min/d)	8.4 ± 11.8	7.5 ± 11.3	9.2 ± 12.2	0.120
Commuting to and from school (min/d)	18 ± 16.3	18.8 ± 16.8	17.3 ± 15.8	0.320
Physical activity during recess (min/d)^d^	16 ± 4.2	15.5 ± 4.1	16.5 ± 4.3	**0.016**
Total sedentary behaviour (min/d)	212.3 ± 99.7	220.5 ± 97.5	204.7 ± 101.3	0.087
Electronic media time (min/d)^d^	101.9 ± 52.4	89.5 ± 46.3	113.2 ± 55	**<0.001**
Watching television and videos (min/d)^d^	67.7 ± 33.2	68.2 ± 33.9	67.3 ± 32.6	0.754
Using a computer and playing video games (min/d)	30.9 ± 31.1	19.2 ± 22.3	41.8 ± 34.0	**<0.001**
Using a mobile phone and playing mobile games (min/d)	3.2 ± 8.8	2.1 ± 5.9	4.2 ± 10.8	**0.012**
Listening to music (min/d)	12.9 ± 21.0	15.7 ± 23.2	10.2 ± 18.5	**0.005**
Playing a musical instrument (min/d)	3.1 ± 10.0	4.2 ± 11.5	2.1 ± 8.3	**0.026**
Reading (min/d)^d^	23.4 ± 23.2	25.1 ± 21.8	21.9 ± 24.4	0.145
Writing (min/d)	6.7 ± 12.3	9.7 ± 13.9	3.9 ± 9.9	**<0.001**
Drawing (min/d)	25.5 ± 25.2	33.4 ± 27.0	18.1 ± 20.9	**<0.001**
Doing arts and crafts (min/d)	13.5 ± 20.2	19.5 ± 21.6	8.0 ± 17.1	**<0.001**
Playing board games (min/d)	15.1 ± 18.3	14.5 ± 16.5	15.6 ± 19.8	0.524
Resting (min/d)^e^	10.6 ± 25.6	9.4 ± 24.9	11.6 ± 26.1	0.366
Maximal work load during exercise test (W/kg of body lean mass)^f^	3.7 ± 0.52	3.6 ± 0.50	3.8 ± 0.50	**<0.001**
Maximal work load during exercise test (W/kg of total body mass)^f^	2.9 ± 0.54	2.7 ± 0.46	3.1 ± 0.53	**<0.001**

Basic characteristics in girls and boys are presented as means and standard deviations. P-values <0.05 are bolded.

*Differences in means between girls and boys were analysed with independent samples *t*-test, ^a^Based on Finnish reference values, ^b^All N = 388, Girls N = 178, Boys N = 210, ^c^Includes 90 minutes of physical activity during physical education per week, ^d^N = 467, ^e^N = 464, ^f^N = 465.

### Independent associations of different types of PA with cardiometabolic risk factors

Total PA was inversely associated with cardiometabolic risk score, body fat percentage, insulin, triglycerides, VLDL triglycerides, VLDL cholesterol, LDL cholesterol and HDL triglycerides and directly related to HDL cholesterol adjusted for age and gender (Table 2), total SB and all dietary factors (data not shown). The associations with the cardiometabolic risk score (β = -0.048, p = 0.232) and insulin (β = -0.038, p = 0.380) disappeared and the associations with triglycerides (β = -0.143, p = 0.002) and HDL cholesterol (β = 0.116, p = 0.013) weakened after further adjustment for body fat percentage.

**Table 2:** Associations of physical activities and sedentary behaviours with cardiometabolic risk factors

	**Cardiometabolic risk score**	**Body fat percentage (%)**	**Waist circumference (cm)**^**A**^	**Fasting serum insulin (mU/l)**^**A**^	**Fasting blood glucose (mmol/l)**^**A**^	**Fasting plasma TG (mmol/l)**^**A**^	**Fasting plasma HDL Chol (mmol/l)**^**A**^	**Systolic blood pressure (mmHg)**^**A**^	**Diastolic blood pressure (mmHg)**^**A**^	**Fasting plasma VLDL TG**^**B**^**(mmol/l)**	**Fasting plasma VLDL Chol**^**B**^**(mmol/l)**	**Fasting plasma LDL Chol (mmol/l)**	**Fasting plasma HDL TG**^**B**^**(mmol/l)**
**Variable**	**β**	**β**	**β**	**β**	**β**	**β**	**β**	**β**	**β**	**β**	**β**	**β**	**β**
**Total physical activity (min/d)**	-0.135**	-0.155***	-0.074	-0.099**	0.02	-0.166***	0.144**	-0.042	0.021	-0.230***	-0.168**	-0.094**	-0.149**
Unstructured physical activity (min/d)	-0.123**	-0.099**	-0.042	-0.108**	-0.011	-0.144**	0.126**	0.018	-0.021	-0.233***	-0.199***	-0.074	-0.089
Structured exercise (min/d)	-0.026	-0.058	-0.026	-0.061	0.029	0.005	0.044	-0.056	0.065	-0.015	0.036	-0.018	0.004
Organised sports (min/d)	-0.042	-0.131**	-0.059	-0.010	0.043	-0.089	0.113**	0.027	0.104**	-0.074	-0.056	-0.092**	-0.086
Commuting to and from school (min/d)	-0.037	-0.030	-0.017	0.024	0.033	-0.083	0.031	-0.086	-0.006	-0.084	-0.042	-0.005	-0.153**
Physical activity during recess (min/d)	-0.098**	-0.148**	-0.107**	-0.097**	-0.064	-0.015	-0.078	-0.14**	-0.096**	0.015	0.039	-0.065	0.016
**Total sedentary behaviour (min/d)**	0.086	0.072	0.052	0.022	0.014	0.130**	-0.074	0.058	-0.038	0.079	0.077	0.077	0.089
Electronic media time (min/d)	0.129**	0.082	0.091	0.089	0.020	0.105**	-0.087	0.061	0.045	0.063	0.075	0.039	0.032
Watching television and videos (min/d)	0.135**	0.090**	0.097**	0.085	0.066	0.065	-0.063	0.096**	0.071	0.014	0.023	0.045	0.021
Using a computer and playing video games (min/d)	0.078	0.032	0.038	0.063	-0.021	0.116**	-0.085	-0.018	0.006	0.107	0.115**	0.043	0.016
Using a mobile phone and playing mobile games (min/d)	-0.020	0.028	0.028	-0.009	-0.074	-0.006	0.010	0.040	-0.036	-0.035	-0.032	-0.081	0.048
Listening to music (min/d)	-0.035	-0.038	-0.056	-0.025	0.032	0.076	0.092**	-0.017	-0.082	0.050	0.019	-0.018	0.141**
Playing a musical instrument (min/d)	-0.071	-0.072	-0.051	-0.134**	-0.015	-0.045	0.004	0.043	-0.050	-0.040	-0.020	-0.012	-0.010
Reading (min/d)	0.036	0.019	0.015	-0.014	0.030	0.048	-0.051	0.027	-0.036	-0.014	-0.020	0.038	0.069
Writing (min/d)	0.030	0.054	0.042	0.001	-0.005	0.031	-0.025	0.049	-0.028	0.000	-0.020	0.059	-0.008
Drawing (min/d)	0.055	0.063	0.031	0.018	0.028	0.066	-0.033	0.05	-0.018	0.037	0.038	0.085	0.028
Doing arts and crafts (min/d)	0.015	0.023	-0.026	0.035	0.028	0.049	0.008	0.025	-0.073	0.066	0.052	0.034	0.072
Playing board games (min/d)	-0.050	-0.014	-0.035	-0.065	-0.027	-0.036	-0.031	-0.033	-0.045	-0.046	-0.048	-0.019	-0.025
Resting (min/d)	0.092**	0.075	0.076	0.035	-0.002	0.131**	-0.086	0.007	-0.011	0.134**	0.147**	0.105**	0.061

Standardized regression coefficients (β) are from linear regression models adjusted for age and gender. Values with p < 0.05 are bolded.

***p < 0.001, **p < 0.01 and *p < 0.05, ^A^Components of cardiometabolic risk score, ^B^N = 388.

Unstructured PA was inversely associated with the cardiometabolic risk score, body fat percentage, insulin, triglycerides, VLDL triglycerides and VLDL cholesterol and directly related to HDL cholesterol adjusted for age and gender (Table 2), total SB and all dietary factors (data not shown). The associations with the cardiometabolic risk score (β = -0.067, p = 0.096) and insulin (β = -0.069, p = 0.114) were no longer statistically significant after controlling for body fat percentage.

Organised sports were inversely associated with body fat percentage and LDL cholesterol and directly related to HDL cholesterol and diastolic blood pressure adjusted for age and gender (Table 2), total SB and all dietary factors (data not shown). The associations with LDL cholesterol (β = -0.069, p = 0.133) and HDL cholesterol (β = 0.088, p = 0.053) were no longer statistically significant after further adjustment for body fat percentage.

PA during recess was inversely related to the cardiometabolic risk score, body fat percentage, waist circumference, insulin and systolic and diastolic blood pressure adjusted for age and gender (Table 2), total SB and all dietary factors (data not shown). The associations with the cardiometabolic risk score (β = -0.014, p = 0.724) and insulin (β = -0.039, p = 0.363) disappeared after additional adjustment for body fat percentage.

PA during commuting to and from school was inversely associated with HDL triglycerides adjusted for age and gender (Table 2), total SB, all dietary factors and body fat percentage (data not shown).

### Independent associations of different types of SB with cardiometabolic risk factors

Total SB was directly associated with triglycerides adjusted for age and gender (Table 2), total PA, all dietary factors and body fat percentage (data not shown).

EMT was directly associated with the cardiometabolic risk score and triglycerides adjusted for age and gender (Table 2). The association with the cardiometabolic risk score remained almost similar after adjustment for total PA (data not shown), but it weakened after controlling for body fat percentage (β = 0.083, p = 0.040) and the number of meals (β = 0.114, p = 0.018). The relationship to triglycerides was no longer statistically significant after adjustment for total PA (β = 0.080, p = 0.094), the number of meals (β = 0.093, p = 0.056) or body fat percentage (β = 0.092, p = 0.051).

Watching TV and videos was directly related to the cardiometabolic risk score, body fat percentage, waist circumference and systolic blood pressure adjusted for age and gender (Table 2). The association with cardiometabolic risk score was similar after further adjustment for total PA (data not shown), but it weakened after controlling for body fat percentage (β = 0.085, p = 0.032) or the number of meals (β = 0.119, p = 0.010). The relationship to body fat percentage remained similar after further adjustment for all dietary factors (data not shown), but it was no longer statistically significant after controlling for total PA (β = 0.077, p = 0.076). The association with waist circumference was similar after further adjustment for total PA (data not shown), but it was no longer statistically significant after controlling for the number of meals (β = 0.083, p = 0.071). The relationship to systolic blood pressure remained similar after adjustment for total PA and all dietary factors, but it was no longer statistically significant after controlling for body fat percentage (β = 0.075, p = 0.097).

Using a computer and playing video games was directly associated with triglycerides and VLDL cholesterol adjusted for age and gender (Table 2) as well as body fat percentage (data not shown). The association with triglycerides was no longer statistically significant after adjustment for total PA (β = 0.075, p = 0.097) or the number of meals, sugar-sweetened beverages and salty snacks (β = 0.082, p = 0.088). Also the association with VLDL cholesterol did not remain statistically significant after adjustment for total PA (β = 0.086, p = 0.120) or the number of meals, sugar-sweetened beverages and salty snacks (β = 0.095, p = 0.091).

Resting was directly associated with the cardiometabolic risk score, triglycerides, VLDL triglycerides, VLDL cholesterol and LDL cholesterol adjusted for age and gender (Table 2) as well as total PA and dietary factors (data not shown). The association with the cardiometabolic risk score was no longer statistically significant after controlling for body fat percentage (β = 0.049, p = 0.215).

Listening to music was directly related to HDL cholesterol and HDL triglycerides adjusted for age and gender (Table 2) as well as total PA, all dietary factors and body fat percentage (data not shown). Playing a musical instrument was inversely associated with insulin adjusted for age and gender (Table 2) as well as total PA and all dietary factors (data not shown). This relationship weakened after adjustment for body fat percentage (β = -0.106, p = 0.014).

### Combined associations of total PA and EMT with the cardiometabolic risk score

**Figure 1:**
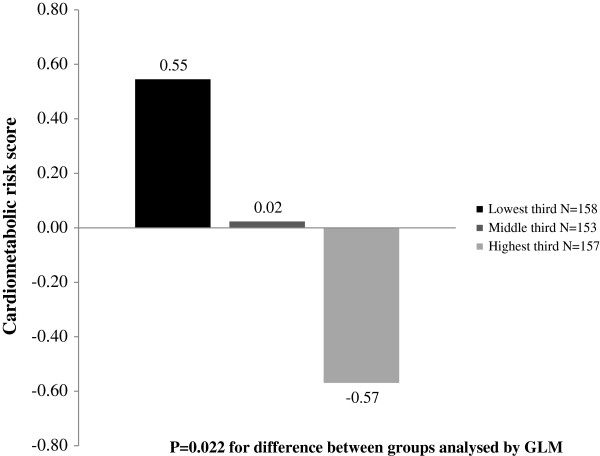
**Means of cardiometabolic risk score in thirds of total physical activity.** Means of cardiometabolic risk score in lowest (<87 min/day), middle (87–127 min/day) and highest (> 127 min/day) third of total physical activity adjusted for age and gender.

**Figure 2:**
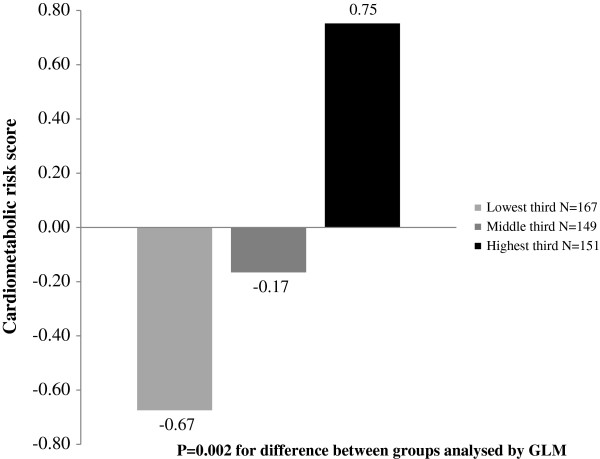
**Means of cardiometabolic risk score in thirds of electronic media time.** Means of cardiometabolic risk score in lowest (<77 min/day), middle (77–115 min/day) and highest (> 115 min/day) third of electronic media time adjusted for age and gender.

**Figure 3:**
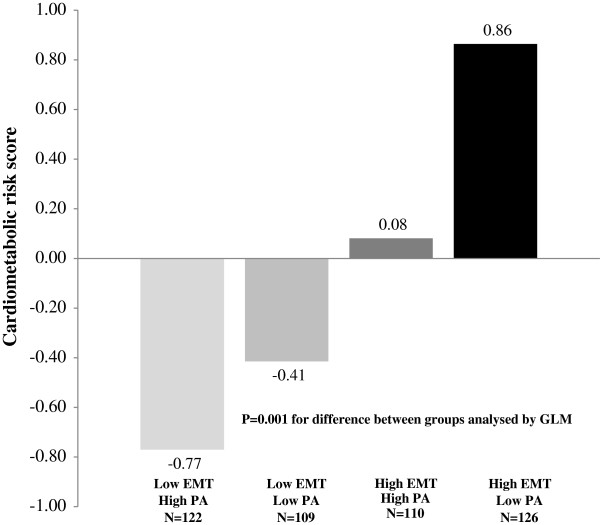
**Combined association physical activity and electronic media time with cardiometabolic risk score.** Combined association of total physical activity (categorised at median of 107 min/day) and electronic media time (categorised at median of 96 min/day) with cardiometabolic risk score adjusted for age and gender.

The cardiometabolic risk score decreased with increasing thirds of total PA after controlling for age and gender (Figure 1). Also body fat percentage (21.0, 19.8 and 18.6%, P = 0.023), VLDL triglycerides (0.31, 0.29 and 0.25 mmol/l, P = 0.001), VLDL cholesterol (0.14, 0.13 and 0.11 mmol/l, P = 0.025) and HDL triglycerides (0.16, 0.16 and 0.15 mmol/l, P = 0.043) decreased and HDL cholesterol (1.54, 1.61 and 1.64 mmol/l, P = 0.019) increased with increasing thirds of total PA. The cardiometabolic risk score increased with increasing thirds of EMT after controlling for age and gender (Figure 2). Whereas the cardiometabolic risk score was highest among children with higher levels of EMT (above median) and lower levels of total PA (below median) after adjustment for age and gender, it was lowest among children with a lower EMT and a higher total PA (Figure 3).

## Discussion

We found that lower levels of total PA, particularly unstructured PA, and higher levels of SB, especially watching TV and videos and resting, were associated with an increased cardiometabolic risk. The combination of lower levels of total PA and higher levels of EMT was related to the highest cardiometabolic risk.

There are a number of studies on the associations of PA and SB with overall cardiometabolic risk and overweight among children [12,13,16-20]. However, few studies have addressed the question, whether PA and SB are related to overall cardiometabolic risk and body adiposity independent of other health behaviours or individual cardiometabolic risk factors in children [27]. In the European Youth Heart Study among children and adolescents, PA was not associated with body adiposity, but it was inversely related to overall cardiometabolic risk independent of watching TV and body adiposity [27]. However, watching TV was directly related to body adiposity and the direct association of watching TV with overall cardiometabolic risk was partly accounted for body adiposity and the frequency of meals. In contrast to the results of the European Youth Heart Study [27], our findings suggest that the inverse association of PA with overall cardiometabolic risk is largely explained by body adiposity. Consistent with the results of the European Youth Heart Study, however, the direct relationship of SB with overall cardiometabolic risk was partly accounted for body adiposity and the number of meals. We also observed that the direct association of watching TV with body adiposity was partly explained by total PA. These findings together suggest that some unhealthy eating behaviours accumulate in children with higher levels of SB.

We found that total, unstructured and recess PA were inversely associated with fasting insulin independent of total SB, but the relationship between total PA and fasting insulin was largely explained by body adiposity. These results are consistent with the observation that PA improves insulin sensitivity by reducing body adiposity [28]. PA has been inversely associated and SB has been directly related to fasting insulin regardless of body adiposity among children in some earlier studies [29-31]. In the European Youth Heart Study among children and adolescents, PA was inversely associated with fasting insulin even after controlling for watching TV and body adiposity, but the direct relationship between watching TV and fasting insulin was explained by body adiposity [27].

Our findings suggest that lower levels of total and unstructured PA and higher levels of total SB and resting are most consistently associated with unfavourable levels of lipids and lipoproteins and that most of these relationships were independent of eating behaviours and body adiposity. PA has been inversely associated with triglycerides and LDL cholesterol and directly related to HDL cholesterol in some studies among children [27,32-35]. However, SB has not been independently associated with triglycerides, LDL cholesterol or HDL cholesterol among children. Moreover, there are no previous studies on the relationships of PA or SB to VLDL triglycerides, VLDL cholesterol or HDL triglycerides among children. PA could decrease triglycerides and increase cholesterol in HDL particles by decreasing triglycerides in lower-density lipoproteins and by changing the activities of lipoprotein lipase, hepatic lipase or lipid transfer proteins [36,37].

We found that only PA during recess was inversely associated with systolic and diastolic blood pressure among children. Organised sports were directly associated with diastolic blood pressure, although they were inversely related to body adiposity. Moreover, watching TV and videos was directly associated with systolic blood pressure. One explanation for our observations is that recess PA may relief emotional stress and thereby reduce sympathetic activity and lower blood pressure and that organised sports and watching TV and videos may have opposite effects among children. In previous studies, evidence on the association of PA with blood pressure among children has been limited and inconclusive [38].

Children in our study sample spent on average six hours per week in unstructured PA and two hours per week in PA during recess. Both of these common types of PA were inversely associated with cardiometabolic risk, suggesting that an increase in unstructured and recess PA could reduce cardiometabolic risk at the population level. Children in our study sample spent almost two hours per day watching TV and videos and with other electronic media. EMT was also directly related to cardiometabolic risk. Therefore, more attention should be paid to the reduction of screen time, particularly if it tends to reduce time spent in PA.

The strengths of our study include a rather large population sample of healthy girls and boys and comprehensive and detailed assessments of cardiometabolic risk factors and possible confounding factors. We used a continuous cardiometabolic risk score, because it is a more sensitive way to describe cardiometabolic risk than dichotomous definitions for metabolic syndrome [14,15,39,40]. Lack of an objective measure of PA is a limitation in our study. Total PA measured by The PANIC Physical Activity Questionnaire had a moderate positive correlation with total PA measured objectively by the Actiheart monitor in a subset of the children, however, suggesting that our questionnaire can be used for the assessment of total physical activity among children. Moreover, we designed the questionnaire specifically for investigating the different types of PA and SB among primary school children. A weakness of our study is the cross-sectional study design that did not allow us to draw conclusions about causality or temporal order of the relationships of PA and SB with cardiometabolic risk. Moreover, although we had an opportunity to control for several confounding factors, we could not rule out residual confounding due to unmeasured or inadequately measured factors.

## Conclusions

The results of our study emphasise increasing total and unstructured PA, decreasing watching TV and videos and other sedentary behaviours and avoiding unhealthy eating to reduce cardiometabolic risk among children.

## Abbreviations

PA: Physical activity; SB: Sedentary behaviour; EMT: Electronic media time.

## Competing interests

The authors declare that they have no competing interests.

## Authors’ contributions

JV participated in the collection of data, conducted the statistical analyses and drafted the manuscript. AME, AV, TT, NL, PK, EKL, JA and HML participated in the data collection and drafted the manuscript. DEL helped in the data analysis and drafted the manuscript. VL helped in obtaining funding, participated in the data collection and drafted the manuscript. TAL planned and conceived the study, was responsible for obtaining funding and lead drafting of the manuscript. All authors read and approved the final manuscript.
